# Bioprospecting Fluorescent Plant Growth Regulators from Arabidopsis to Vegetable Crops

**DOI:** 10.3390/ijms22062797

**Published:** 2021-03-10

**Authors:** Radu L. Sumalan, Liliana Halip, Massimo E. Maffei, Lilia Croitor, Anatolii V. Siminel, Izidora Radulov, Renata M. Sumalan, Manuela E. Crisan

**Affiliations:** 1 Faculty of Horticulture and Forestry, Banat’ s University of Agriculture Science and Veterinary Medicine “King Michael Ist of Romania” from Timisoara, Calea Aradului nr 119, 300645 Timisoara, Romania; sumalanagro@yahoo.com (R.L.S.); srenata_maria@yahoo.com (R.M.S.); 2 “Coriolan Drăgulescu” Institute of Chemistry, 24 Mihai Viteazul Blvd., 300223 Timisoara, Romania; 3 Department Life Sciences and Systems Biology, University of Turin, Via G. Quarello 15/a, 10135 Turin, Italy; massimo.maffei@unito.it; 4 Institute of Applied Physics, Academiei Street 5, MD2028 Chisinau, Moldova; croitor.lilia@gmail.com (L.C.); ansi@inbox.ru (A.V.S.); 5 Faculty of Agriculture, Banat’s University of Agriculture Science and Veterinary Medicine “King Michael Ist of Romania” from Timisoara, Calea Aradului nr 119, 300645 Timisoara, Romania; isidoraradulov@yahoo.com

**Keywords:** auxin, ethanolammonium *p*-aminobenzoate, fluorescence, Arabidopsis, cucumber, tomato, Hirshfeld surfaces, homology model, molecular docking, auxin receptor TIR1

## Abstract

The phytohormone auxin is involved in almost every process of a plant’s life, from germination to plant development. Nowadays, auxin research connects synthetic chemistry, plant biology and computational chemistry in order to develop innovative and safe compounds to be used in sustainable agricultural practice. In this framework, we developed new fluorescent compounds, ethanolammonium *p*-aminobenzoate (HEA-*p*ABA) and *p*-nitrobenzoate (HEA-*p*NBA), and investigated their auxin-like behavior on two main commercial vegetables cultivated in Europe, cucumber (*Cucumis sativus*) and tomato (*Solanum* *lycopersicum*), in comparison to the model plant Arabidopsis (*Arabidopsis thaliana*). Moreover, the binding modes and affinities of two organic salts in relation to the natural auxin indole-3-acetic acid (IAA) into TIR1 auxin receptor were investigated by computational approaches (homology modeling and molecular docking). Both experimental and theoretical results highlight HEA-*p*ABA as a fluorescent compound with auxin-like activity both in Arabidopsis and the commercial cucumber and tomato. Therefore, alkanolammonium benzoates have a great potential as promising sustainable plant growth stimulators to be efficiently used in vegetable crops.

## 1. Introduction

Since its discovery nearly a century ago, deciphering the complexity of auxin action has been an open question in plant hormone research [1]. In the last decades, the interconnection between modern biology and chemistry has led to progress in plant growth regulators and herbicides, auxins being perceived as small molecules with a big impact [2]. Auxins act as signaling molecules to regulate almost every aspect of plant growth and developmental processes [3,4], being involved in interactions with other hormone signals [5] and even with microorganisms and viruses [6]. Specifically, indole-3-acetic acid (IAA), the most abundant naturally occurring auxin, is involved in promotion of hypocotyl and inhibition of main root elongation, the induction of lateral root formation and gravitropism [7,8].

The synthesis of small bioactive compounds with auxin-like activity has gained increasing attention in plant chemical biology research as a valuable tool to study and control molecular features of auxin action, as well as in agricultural and horticultural practices by controlling plant growth. The extensive bioassay investigations used for generating auxin structure–activity relationships highlighted two essential features of the molecule responsible for its biological activity: a planar aromatic ring structure and a carboxyl group [8]. Moreover, the side groups binding to the ring structure can dictate the activity, adding a level of specificity [9].

To mitigate the negative effects of chemicals on the environment, recent European strategies require serious restrictions on various compounds used in crop production that contain synthetic auxins and plant protection chemicals. Therefore, the discovery of new sustainable auxin-related compounds for agronomically interesting species is essential. Small biologically active molecules, such as benzoic acid and its derivatives, which are important structural elements for many drugs and natural products, drew attention in the last years due to their involvement in various plant physiological processes [10]. Beside the presence of carboxylate and a planar aromatic ring, the substituent on the benzene ring exhibits an essential role in biological activity establishment. Our previous studies revealed the important role of different substituted benzoic acids in the synthesis of stable acid–base systems with supramolecular architectures guided by hydrogen bonding interactions [11,12,13,14,15], low toxicity [16] and promising plant growth regulatory activity on the model plant Arabidopsis (*Arabidopsis thaliana*) [17,18] and the vegetable crops cucumber (*Cucumis sativus*) [19] and tomato *(Solanum lycopersicum*) [20,21].

The crystallographic determination of transport inhibitor response 1 (TIR1) [22], as an auxin receptor, brings fundamental information to reveal the mechanism of auxin action. The protein receptor recognizes both the naturally occurring IAA and synthetic auxins such as 1-naphthaleneacetic acid (NAA) and 2,4-dichlorophenoxyacetic acid (2,4-D). Moreover, the interactions responsible for molecular recognition indicate a higher affinity for IAA [23]. The *Arabidopsis* model is frequently used in auxin research because it is the best-characterized dicotyledonous plant species in terms of regulation of molecular mechanisms for growth and development.

Recently, fluorescence labeling of auxin molecules has drawn attention as a successful strategy to investigate the transport, perception and mode of action by visualizing their plant location and distribution as well in the identification of the complex ligand-receptor [24]. The literature mentions few fluorescent auxin compounds: fluorescein isothiocynate and rhodamine isothiocynate conjugates of IAA (IAA-FITC, IAA-RITC) [25,26] as well as nitrobenzoxadiazole (NBD)-labeled IAA, NAA and 2,4-D analogues [27,28].

Here we report the development of new fluorescent compounds, ethanolammonium *p*-aminobenzoate (HEA-*p*ABA) and *p*-nitrobenzoate (HEA-*p*NBA), and investigate their auxin-like plant growth regulatory activity on two main commercial vegetables cultivated in Europe, cucumber and tomato. The biological activities were analyzed and discussed as related to Arabidopsis model plant responses, the results being compared with our earlier research [18]. The new compounds with dual biological activity were obtained based on biologically active components (anion and cation) with well-known toxicological properties [16] or natural metabolites, accepted as safe compounds. *p*-Amino- and *p*-nitrobenzoic acids (*p*ABAH, *p*NBAH) are commercially available reagents which received increasing attention as building blocks in drug development [29,30] as well as in plant science [18,21], *p*ABAH being a precursor of folic acid [31]. Ethanolamine (EA) is an important metabolite in plants and is a precursor of choline and membrane lipids [32]. Computational approaches such as homology modeling and molecular docking were used to explain the auxin-like behavior of these compounds against TIR1 in cucumber and tomato.

## 2. Results and Discussions

### 2.1. Bioactivity Assays

The response of seed germination and root growth development (primary root length and lateral root number) to HEA-*p*ABA, HEA-*p*NBA and natural auxin IAA used as reference was investigated in Arabidopsis, cucumber and tomato. The compounds were tested in different concentrations, using 0.1 mM as the starting point for experiments, as a result of our previous screening [18]. The data showed that exogenous treatments inhibited the seed germination in all studied species, when compared to control (Table 1), IAA showing the significant inhibitory effect and HEA-*p*ABA the lowest one. At 0.5 mM and 1 mM concentrations, HEA-*p*NBA treatment reduced the germination by about 10%, whereas IAA caused an almost complete inhibition of Arabidopsis and tomato seed germination (Table 1).

The tomato seeds treated with 0.5 mM IAA began to germinate only after 14 days (data not shown). At 0.1 mM concentration, HEA-*p*ABA and HEA-*p*NBA treatments highlighted differences in seed germination even after five days, the tenth day revealing an uniformization tendency of values. Despite this, IAA showed the slowest germination process which started on the fifth day both in Arabidopsis and tomato.

Recent studies have reported that exogenous IAA application higher than 1 μM determines the inhibition or delay of seed germination in plants [33,34]. However, the mechanism of auxin action in seed germination has not been completely elucidated, although there are studies which identified the key role of interactions between IAA, abscisic acid (ABA) and even gibberellic acid (GA) to control the seed dormancy and germination [35,36].

**Figure 1:**
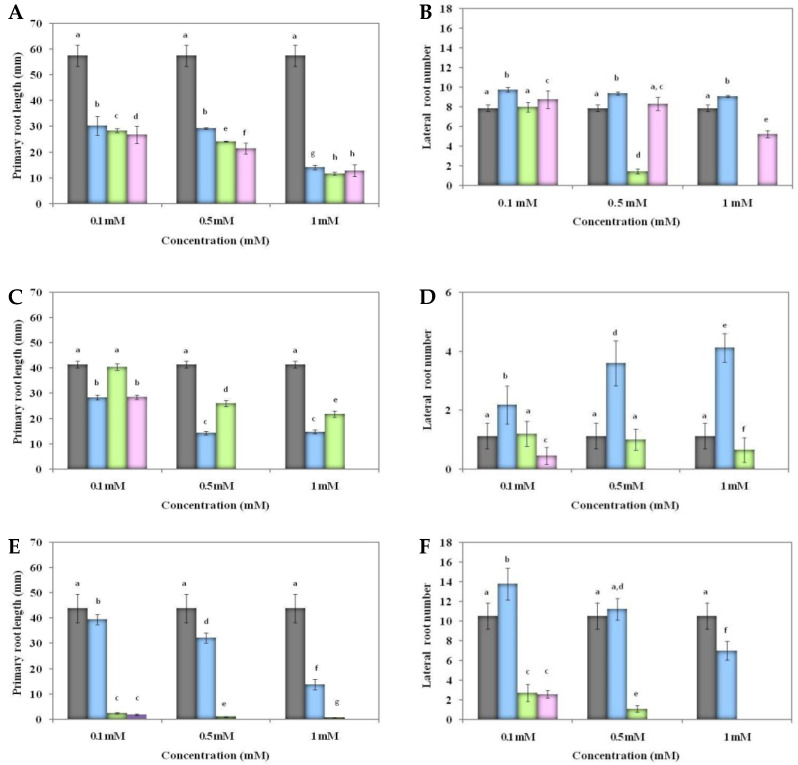
Effect of HEA-*p*ABA (ethanolammonium *p*-aminobenzoate; blue), HEA-*p*NBA (ethanolammonium *p*-nitrobenzoate; green), IAA (indole-3-acetic acid; pink) different concentrations and control (grey) on primary root length and lateral root number of *C. sativus* (**A**,**B**), *S. lycopersicum* (**C**,**D**) and *A. thaliana* (**E**,**F**). In all figures, different letters represent statistically Scheme 0. Metric bars indicate the standard error of the mean (SEM) of 5 repeated measurements.

Besides involvement in seed germination, auxins play an essential role in plant development, especially in root growth, by inhibiting the primary root length and increasing the lateral root number. We evaluated these parameters in order to investigate the role of HEA-*p*ABA and HEA-*p*NBA on early root development. The effect of treatments on root growth was evaluated by using different concentrations ranging between 0.1 mM and 1 mM (Figure S1, Supplementary Materials). Significant differences were detected both between the various concentrations of each treatment as well as between treatments. In particular, HEA-*p*ABA, HEA-*p*NBA and IAA treatments suppressed the cucumber primary root length in a concentration-dependent manner (Figure 1A), whereas a stimulation of cucumber lateral root number was observed with HEA-*p*ABA treatment, HEA-*p*NBA having an opposite effect upon 0.5 mM and 1 mM treatment (Figure 1B). In tomato, IAA showed the same inhibitory effect as HEA-*p*ABA on primary root length when used at 0.1 mM and completely inhibited the primary root length when used at 0.5 mM and 1 mM (Figure 1C). The lateral root number of tomato was significantly (*p* < 0.05) increased by HEA-*p*ABA at all concentrations, whereas IAA (at all concentrations) and HEA-*p*NBA (at 1 mM) had an opposite effect (Figure 1D). Finally, Arabidopsis primary root length was strongly inhibited by IAA and HEA-*p*NBA at all concentrations, whereas HEA-*p*ABA showed a reduced inhibition (Figure 1E). With regards to lateral root number, Arabidopsis showed almost the same trend as observed for tomato; however, HEA-*p*ABA strongly promoted lateral roots only at 0.1 mM (Figure 1F).

In tomato and Arabidopsis, IAA treatment induced a strong inhibition both of the primary root length and lateral root number. These results support the observation that the promotion of lateral root number gradually decreases with the reduction of root cell elongation when IAA acts at high concentrations [37]. Thus, a correlation between root cell elongation, concentration level of IAA and lateral root number was evidenced. Moreover, it is known that IAA cannot reduce cell length in full elongated root portions [37]. Our previous results on tomato confirm a longer time root development in the case of IAA seedlings treatment, revealing an auxin-like activity after 21 days treatment in greenhouse [20]. Instead, HEA-*p*ABA activity results suggest an earlier promoting root development compared with IAA. In conclusion, HEA-*p*ABA shows a potential auxin-like root growth phenotype, with primary root length being inhibited, while lateral root number was stimulated at all concentrations in all plants studied. 

### 2.2. Fluorescence Properties

**Figure 2:**
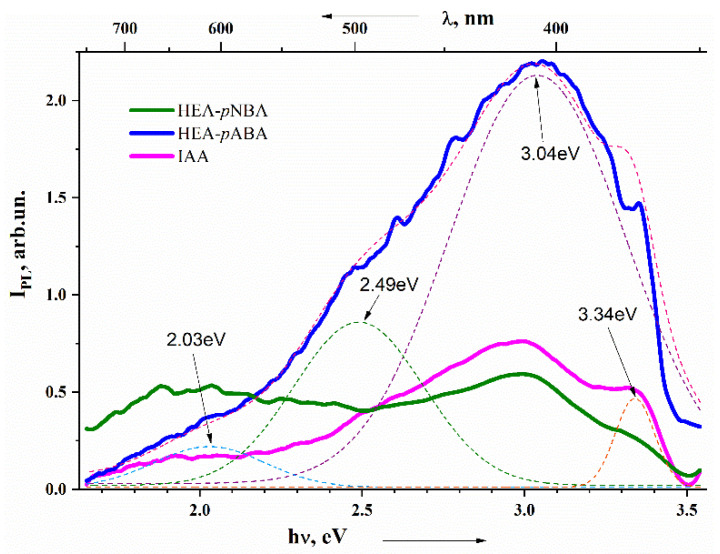
Emission spectra of HEA-*p*ABA and HEA-*p*NBA versus IAA. The deconvolution of Gaussian distribution functions are shown by thin dotted lines with the presentation of the maximum peaks in eV.

The fluorescence for the organic salts HEA-*p*NBA and HEA-*p*ABA, as well as for IAA was studied in the solid state upon excitation at 337 nm (Figure 2).

The Gaussian distribution method was used for spectra division into separate bands, making it possible to distinguish four bands in each spectrum with peaks at 2.03 eV (610 nm), 2.49 eV (498 nm), 3.04 eV (407 nm) and 3.34 eV (371 nm). These bands reveal orange, green, violet and ultraviolet luminescence and are developed in all three spectra with various intensities and with same half width maximum values. The highest luminescence intensity was shown by the 3.04 eV band in the HEA-*p*ABA sample, emitting violet light. As can be seen, this band also has the highest intensity in IAA. The same band (3.04 eV) in the HEA-*p*NBA sample has an equal intensity with the band at a maximum of 2.03 eV (exhibiting orange light) and lower luminescence intensity than in the other two samples, which may explain the change in the properties of this compound in comparison with the others. A lower luminescence intensity may mean that, in this compound, the indicated vibrational modes are exchanged for phonons (i.e., they are quenched), which usually results in a lower activity of the sample. The results indicate that modification of the fluorescence property in the case of studied organic salts was generated by the presence of different substituent groups on the benzene ring.

Overall, HEA-*p*ABA provided a higher fluorescence than IAA as well as potential auxin-like activity in Arabidopsis and in the two main commercial vegetables, cucumber and tomato, properties which should be considered for future design of new fluorescent plant growth regulators.

### 2.3. Hirshfeld Surfaces

The opposite behavior exhibited by the two organic salts is intriguing since both compounds possess the molecular features responsible for the auxin activity, i.e., planar aromatic ring and carboxylate function. The overlay of the phenyl carbon atoms of HEA-*p*NBA and HEA-*p*ABA illustrates their conformational similarity and differences (Figure S2, Supplementary Materials). Both anions form practically planar systems in discussed organic salts, as the least-square plane of the phenyl ring and the least-square plane of the COO^‒^ and NO_2_ groups are 5.81 and 4.81° in HEA-*p*ABA, respectively, and the dihedral angle between the least squares plane of the phenyl ring C6 and the COO‒ group in HEA-*p*ABA is equal to 5.79°.

Surface characteristics and quantitative investigation of intermolecular interactions in the crystal packing of HEA-*p*NBA and HEA-*p*ABA organic salts was done by the analysis of Hirshfeld surfaces maps and two-dimensional (2D) fingerprint plots. The slightly lower Hirshfeld volume and surface area in HEA-*p*ABA (VH = 251.19 Å, SH = 249.90 Å) indicate a more crowded environment of this salt compared to HEA-*p*NBA organic salt (VH = 257.10 Å, SH = 266.64 Å). Globularity (G) and asphericity (Ω) values of HEA-*p*NBA (G = 0.733, Ω = 0.288) and HEA-*p*ABA (G = 0.770, Ω = 0.344) show that both compounds deviate from spherical surface and symmetry.

**Figure 3:**
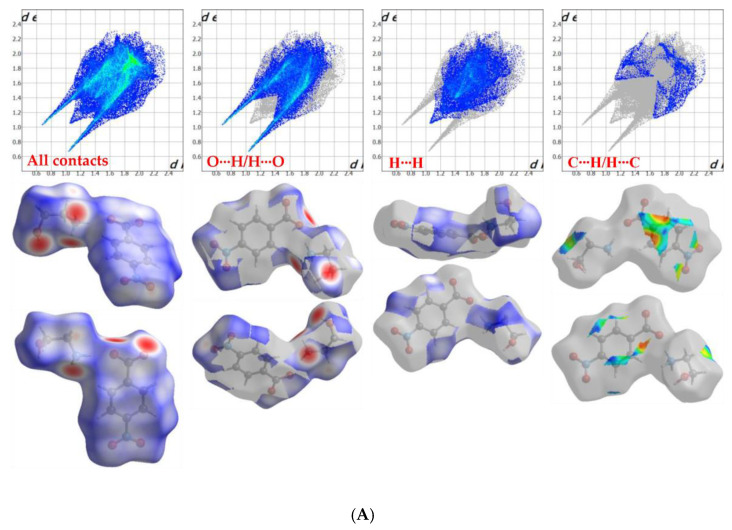

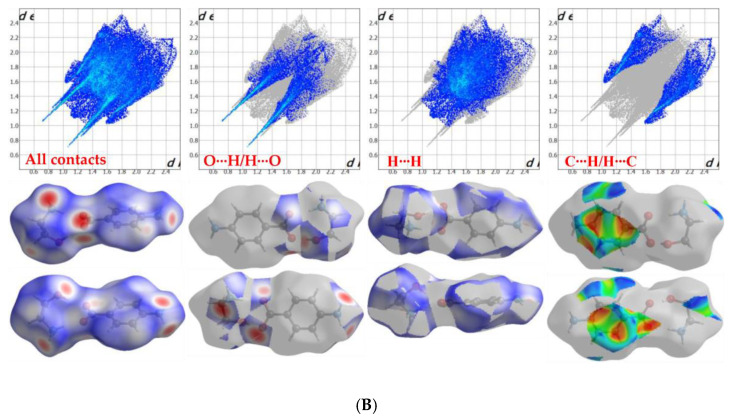
2D fingerprint plots (up) and Hirshfeld surfaces (down) of the different interactions (for all contacts, O∙∙∙H/H∙∙∙O, H∙∙∙H and C∙∙∙H/H∙∙∙C) in both compounds, HEA-*p*NBA (**A**) and HEA-*p*ABA (**B**), in two orientations.

The Hirshfeld surfaces are mapped on the d_norm_ (ranged −0.707 to 1.207 Å for HEA-*p*NBA and −0.632 to 1.290 Å for HEA-*p*ABA) and shape index (−0.989 (concave) to 0.996 (convex) Å for HEA-*p*NBA and −0.997 (concave) to 0.997 (convex) Å for HEA-*p*ABA). The 2D fingerprint plots for HEA-*p*NBA and HEA-*p*ABA correspond to Hirshfeld surfaces and are represented as plots of *di* versus *de*. Figure 3 shows 2D fingerprint plots of Hirshfeld surface for both organic salts and clearly indicates the different distribution of various interactions in the crystal structures. In the HEA-*p*NBA structure is evidenced the predominance of the O∙∙∙H/H∙∙∙O interactions, with a share of 50.5%, while in HEA-*p*ABA prevail the H∙∙∙H contacts (48.9%). In the fingerprint maps the O∙∙∙H/H∙∙∙O interactions are perceived as two extended symmetrical spikes in the middle part of both plots, while the H∙∙∙H contacts appear in the middle of the scattered points.

**Figure 4:**
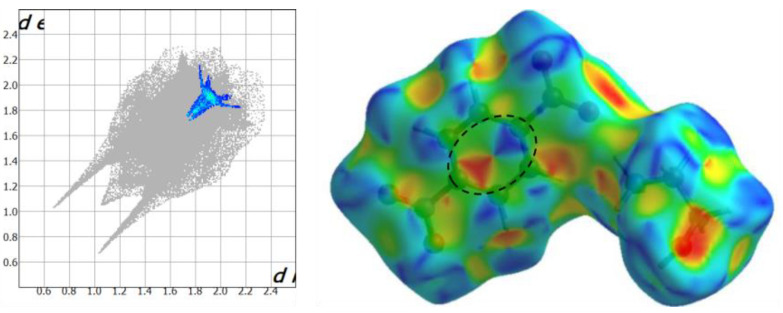
The 2D fingerprint plot (**left**) and shape index (**right**), ranging from −0.989 (red) to 0.996 (blue) Å, of the C∙∙∙C interactions in HEA-*p*NBA.

The next share of entire surface is related to C∙∙∙H/H∙∙∙C contacts (6.6% in HEA-*p*NBA and 22.5% in HEA-*p*ABA) that are observed as two partially wide wing-like peaks and usually represent C‒H∙∙∙π interactions. These contacts prevail in HEA-*p*ABA more than 3 times than in the HEA-*p*NBA structure, while in the last one are observed C∙∙∙C contacts (5.1%) attributed to π∙∙∙π interactions, which do not appear in HEA-*p*ABA. The inspection of C∙∙∙C interactions in HEA-*p*NBA shows a clear signature on the 2D fingerprint plot (Figure 4), which contains a green/blue triangle region (bow-tie pattern) or can be observed as red and blue triangles on the shape-index surface.

**Figure 5:**
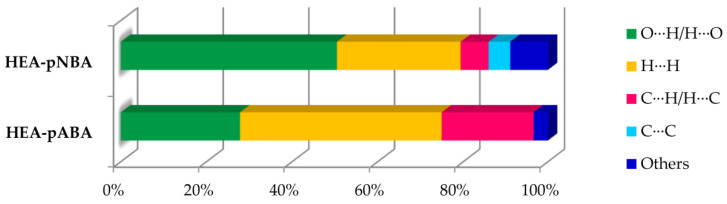
Relative contributions of various intermolecular contacts to the Hirshfeld surface area in HEA-*p*NBA and HEA-*p*ABA discussed in detail in the text.

The relative contributions of the intermolecular contacts exhibited by both organic salts are depicted in Figure 5 which clearly shows that the other interactions are minimal in HEA-*p*ABA (only 3.5% representing N∙∙∙H/H∙∙∙N contacts) compared to 8.9% in HEA-*p*NBA (C∙∙∙N/N∙∙∙C, O∙∙∙O, O∙∙∙N/N∙∙∙O and H∙∙∙N/N∙∙∙H contacts, in decreasing order).

### 2.4. Homology Modeling and Ligand Docking

**Figure 6:**
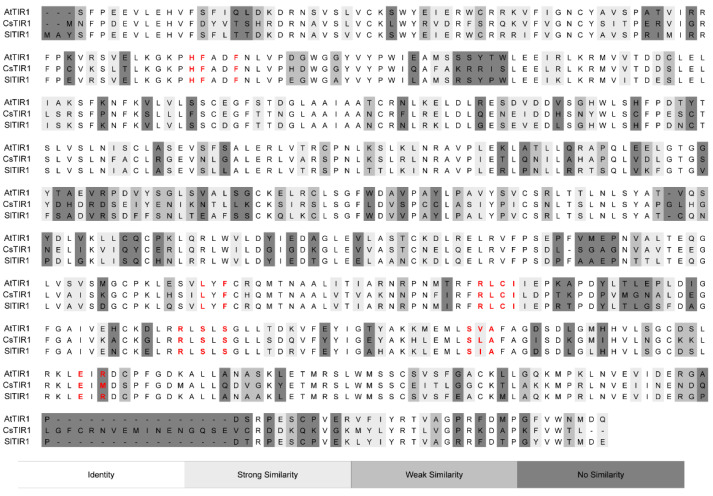
Sequence alignment between AtTIR1, CsTIR1 and SlTIR1. Amino acids are depicted as single-letter codes. Residues from the auxin-binding site are shown in red. The color of background indicates sequence similarity from white (identity) to dark grey (no similarity). See Table S1 in Supplementary Materials for detailed information about sequence similarity.

In order to find an explanation for the contrasting activities of two similar organic salts we proceeded to analyze the interaction of the active component with the auxinic receptor TIR1. As the three-dimensional structures of the TIR1 from cucumber (CsTIR1) and tomato (SlTIR1) have not yet been solved, we used homology modeling to obtain accurate 3D models of these proteins. Homology modeling is a computational method extensively used to predict a 3D structure of a protein from its amino acid sequence relying on the paradigm that similar sequences fold into identical structures. The closest “relative” of CsTIR1 and SlTIR1 with a known 3D structure is the X-ray structure of TIR1 isolated from Arabidopsis (AtTIR1). Therefore we used AtTIR1 to generate 3D models of CsTIR1 and SlTIR1. The sequence alignment reveals that the sequence similarity of CsTIR1 and SlTIR1 with AtTIR1 is very high (over 75%). The similarity increases over 90% if only the binding site is considered (Table S1, Supplementary Materials). The refined alignment (Figure 6) was used to build three-dimensional models of CsTIR1 and SlTIR1 based on AtTIR1.

The homology models of CsTIR1 and SlTIR1 (Figure S3) were sterically and geometrically validated and then were tested for their ability to reproduce the binding mode of IAA in the crystal structure. Considering that AtTIR1, CsTIR and SlTIR1 are highly similar (Table S1), an accurate model would be able to reproduce the crystallized ligand (IAA).

**Figure 7:**
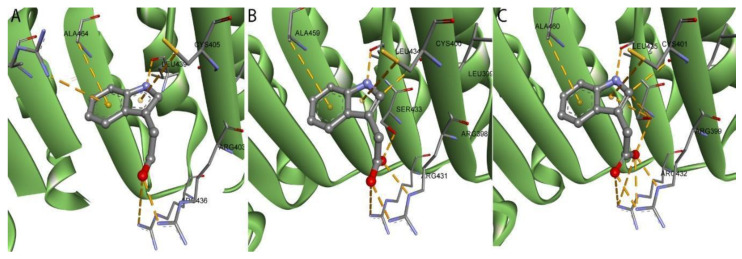
IAA in the AtTIR1 (**A**) CsTIR1 (**B**) and SlTIR1 (**C**) binding site. The secondary structure of the TIR1 is shown in green. IAA is shown as balls and sticks representation, while the amino acids from the binding site are shown with sticks. All interactions are shown as orange dashed lines.

According to experimental data [22], the IAA establishes a complex network of interactions in the AtTIR1-binding site (Table S2). Carboxylate moiety has a proper location and orientation to form hydrogen bonds with Arg403 and Ser438 and a salt bridge with guanidine groups of Arg436 and Arg403. The indole ring position is secured by two opposite π-alkyl interactions formed with Cys405 (pyrrole ring) and Ala464 (phenyl ring). The indole ring also forms a π-cation bond with Arg489 (benzene ring) and a hydrogen bond with the main chain of Leu405 (Figure 7A). Excepting the π-cation bond with Arg489, all the other interactions of IAA are retrieved in the CsTIR1- and SlTIR1-binding sites (Figure 7B,C). The missing interaction does not significantly influence the IAA’s active conformation in the binding site as the indole ring is being maintained in the same position through the interactions with alanine, cysteine and leucine residues. These observations certify that the 3D models of CsTIR1 and SlTIR1 are reliable to be used to observe and explain the behavior of other related ligands.

In order to explain the biological behavior of HEA-*p*ABA and HEA-*p*NBA in previous tests, their anions were docked in the IAA-binding site using Glide. The output conformations were analyzed based on their binding energies with the TIR1 and based population clusters. Additionally, we mapped the auxin-binding site by identifying regions which easily accommodate part of ligands with hydrogen bond acceptors, hydrogen bond donors or hydrophobic areas [38,39].

**Figure 8:**
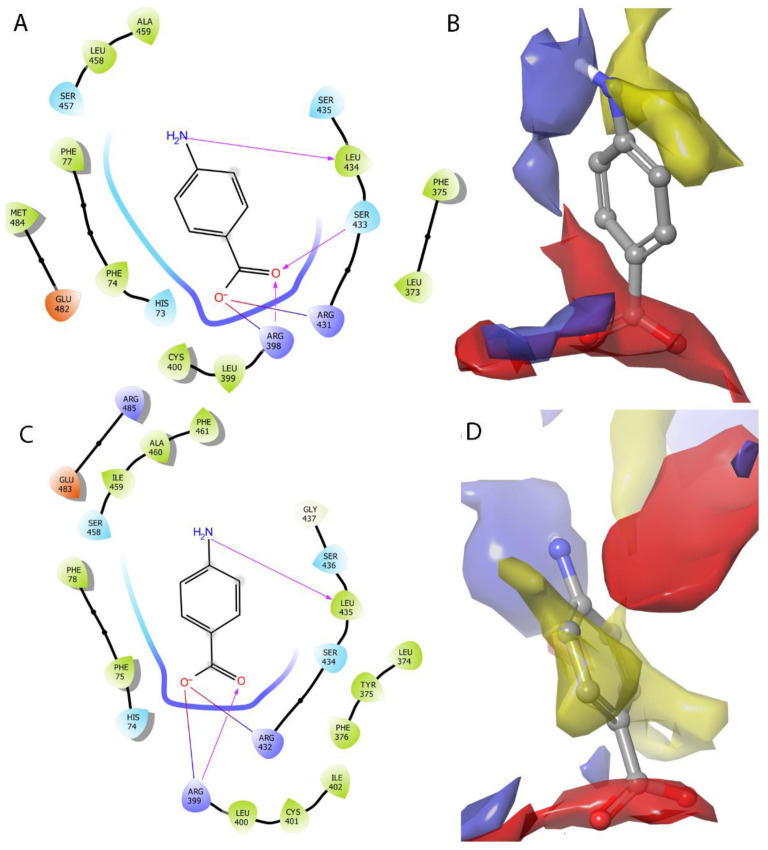
Binding model of HEA-*p*ABA in the auxin-binding site of CsTIR1 (**A**,**B**) and SlTIR1 (**C**,**D**). The blue surface is favorable for occupancy by functional groups that act as hydrogen bond donors, the red surface for hydrogen bond acceptor groups and the yellow areas can easily accommodate hydrophobic parts of a potential ligand.

HEA-*p*ABA has a similar binding mode with IAA in both CsTIR1- and SlTIR1-binding sites. The negatively charged carboxylate interacts with two arginine residues through a salt bridge and/or hydrogen bond formation while the amino group is favorably placed to establish a hydrogen bond with Leu434 in CsTIR1 and Leu435 in SlTIR1 (Figure 8).

**Figure 9:**
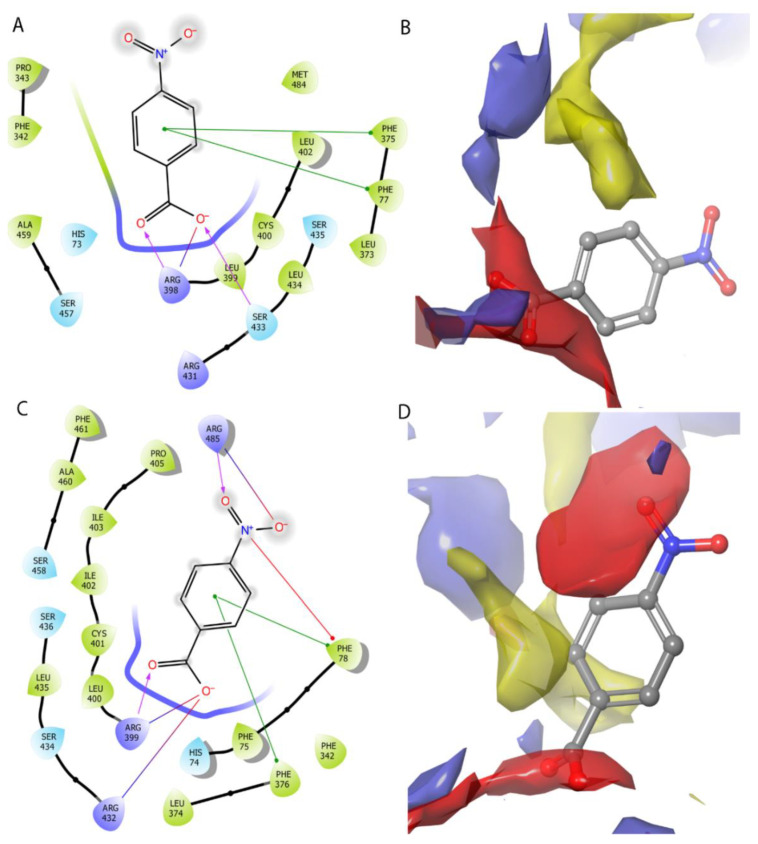
Binding model of HEA-*p*NBA in the auxin-binding site of CsTIR1 (**A**,**B**) and SlTIR1 (**C**,**D**). The blue surface is favorable for occupancy by functional groups that act as hydrogen bond donors, the red surface for hydrogen bond acceptor groups and the yellow areas can easily accommodate hydrophobic parts of a potential ligand.

HEA-*p*NBA adopts a different orientation in the situs: although the carboxylate group forms hydrogen bonds and s salt bridge with the same arginine residues as IAA and HEA-*p*ABA, the ligand HEA-*p*NBA is pushed outside the binding site so that the hydrogen bond with the leucine main chain is not established. This movement is due to the presence of the negatively charged substituent, the benzene ring, which is pushed away from the binding site area which demands a hydrogen bond donor (Figure 9B,D). The hydrogen bond with the Leu439 main chain seems to play an important role in anchoring the ligand in the binding site.

These findings offer an explanation for the different activity of HEA-*p*ABA and HEA-*p*NBA against auxinic receptor TIR1. The active conformation of HEA-*p*ABA shows a perfect fit, both geometrically and electrostatically, in the TIR1-binding site (Figure S4). Even if HEA-*p*NBA has a similar volume and shape, the electrostatic repulsion prevents it from activating the receptor and thus triggering the biological response. All these together indicate correlation with biological tests and explain the auxin-like activity of HEA-*p*ABA in all studied plants by similar binding mode to TIR1 receptor.

## 3. Materials and Methods

### 3.1. Plant Materials, Chemicals and Methods

Seeds of *A. thaliana*, wild-type ecotype Columbia 0 (Col-0), *C. sativus* L. var. Piccolo di Parigi and *S. lycopersicum* L. (Tomin cultivar created by BUASVM Timisoara, Romania) were used. The reagents *p*ABAH, *p*NBAH and EA were of analytical grade and were purchased from Fluka (Basel, Switzerland) and Merck (Darmstadt, Germany), respectively. Compounds HEA-*p*ABA and HEA-*p*NBA were prepared in good yields (90%) by proton exchange reactions, as described earlier [11,18]. Colorless single crystals were obtained by slow evaporation in ethanol solutions at room temperature and their purity (>99%) was established by spectrophotometry [18]. Hirshfeld surface analysis and two-dimensional fingerprint plots were generated by *CrystalExplorer17* (Version 17.5) [40]. The natural auxin IAA was purchased from Sigma-Aldrich (St. Louis, MO, USA) and was used as a positive control for plant growth experiments. Fluorescence emission spectra of single crystals (HEA-*p*ABA, HEA-*p*NBA and IAA) excited with nitrogen laser λex = 337 nm, duration = 15 ns, time repetition 50 Hz were collected at room temperature.

### 3.2. Arabidopsis Assay

The potential auxin-like activity of the compounds was evaluated by the Arabidopsis seedling growth test as previously described [17,18]. Seeds of Arabidopsis Col-0 were surface sterilized with bleach solution (5% (*w*/*v*) Ca(OCl)₂ + 0.02% (*v*/*v*) Triton X-100 in 80% EtOH) for 10 min by continuous shaking, washed 2 times with 80% EtOH, then with 100% EtOH and finally 3 times with sterile distilled water. Square Petri dishes (12 × 12 cm) with 15 seeds and 80 mL modified MS agar medium [41] supplemented with three different concentrations (0.1 mM, 0.5 mM and 1 mM) of tested compounds (HEA-*p*ABA and HEA-*p*NBA, respectively) as well as IAA as a reference control were used. All tested compounds were dissolved in 5 mM MES (2-[*N*′-morpholino] ethanesulfonic acid) buffer (pH 6), sterilized by ultrafiltration (Millipore) and added to MS agar control (∼50 °C). Five repetitions for control and for each tested compound were performed. For breaking dormancy and synchronizing germination, Petri dishes were horizontally stratified for 2 days at 4 °C in the dark and then transferred to the growth chamber at 22 °C and grown in vertical position under 16 h light/8 h dark photoperiod (220 µmol m^−2^s^−1^ photosynthetic photon flux density) for 10 days. 

### 3.3. Cucumber Assay

Cucumber seeds were washed with water for 4 h before germination and then surface sterilized by soaking in 50% (*v*/*v*) NaOCl solution for 10 minutes and rinsed repeatedly with distilled water before transferring them to Petri dishes. For seed germination experiments, 15 healthy and uniformly sized seeds were placed in 9 cm diameter Petri dishes containing two Whatman filter papers moistened with 5 mL sterilized 5 mM MES buffer (pH 6) for control and supplemented with HEA-*p*ABA, HEA-*p*NBA and IAA treatments at three different concentrations (0.1 mM, 0.5 mM and 1 mM). Petri dishes with 15 sterile cucumber seeds were incubated for 3 days at 28 °C in darkness. Five repetitions for control and for each tested compound were performed.

### 3.4. Tomato Assay

The protocols of tomato (surface sterilized) and seed germination were the same as mentioned for cucumber. Each experiment was repeated five times. All plates were placed at 25 ± 2 °C, 70–75% relative humidity under 16 h light/8 h dark photoperiod for 10 days.

### 3.5. Data Processing and Statistical Analysis

The images of Arabidopsis seedlings were acquired after 5 and 10 days by scanning each Petri dish with an EPSON Perfection 4870 Photo scanner at 600 DPI. Germination was defined as radicle emergence by more than 1 mm. Cucumber seedlings were photographed after 3 days, while tomato seedlings after 5 and 10 days. The root lengths were measured using the ImageJ software (Fiji) [42]. The number of emerged lateral roots was counted using a Nikon YS 100 microscope 10× objective. The experimental data were statistically analyzed using a one-way ANOVA, followed by Fisher least significant difference (LSD) test and expressed as the means ± standard error of the mean (SEM). In all plots, different letters indicate significant differences (*p* < 0.05).

### 3.6. Homology Modeling and Refinement

The three-dimensional structures of CsTIR1 and SlTIR1 were built based on a crystal structure of TIR1 from Arabidopsis (AtTIR1, PDB Code: 2P1P) [22] using Prime module from Schrödinger package, release 2020-4 [43,44]. The multiple sequence alignment resulted from Prime was manually refined according to the 3D structure of the AtTIR1, thus deletions or insertions in the *alpha*-helices or *beta*-sheets were avoided. Thirty models of CsTIR1 and SlTIR1 were generated and submitted to the loop refinement step, using the Protein *Refinement* option from the Prime module. The best models were chosen according to their ability to reproduce the conformation of the crystallized ligand and based on energetic criterion. The final models have over 90% of residues in the favorable regions of the Ramachandran map and all the main-chain and side-chain parameters are in the normal range.

### 3.7. Molecular Docking

Ligand setup. Considering that the active component of this type of compound is the anion, only HEA-*p*ABA and HEA-*p*NBA were considered for molecular docking. Ligands (anions) were prepared for docking using LigPrep application from Schrödinger release 2020-4 [45]. The polar and non-polar hydrogen atoms were added to ligands and all possible tautomers at physiological conditions (pH = 7.4) were generated. Furthermore, the ionization states were set and the final geometries were minimized using OPLS_2005 force field.

Protein setup. Protein Preparation Workflow (Schrödinger release 2020-4) [46,47] was used to add all the hydrogen atoms to the CsTIR1 and SlTIR1 receptors, to optimize the charge state of Asp, Glu, Arg, Lys and His residues and the orientation of hydroxyl and amide groups.

Docking protocol. Glide receptor grids were generated from the prepared receptor model, with the docking grids centered on the crystallized ligand. The binding site was defined by a cubic docking box with the dimensions large enough to accommodate ligands with a length ≤20 Å, while to the ligand-midpoint box a side of 14 Å was given. The docking runs were performed with Glide in SP mode using default settings for parameters (Schrödinger release 2020-4) [48,49,50,51].

## 4. Conclusions and Future Perspectives

In recent years, fluorescent auxins have offered understanding of molecular mechanisms of auxin transport, perception and mode of action. Herein, two new compounds, HEA-*p*ABA and HEA-*p*NBA, with higher fluorescence intensity than classical IAA were tested as potential auxin-like plant growth regulators on two main commercial vegetables, cucumber and tomato, as related to model plant Arabidopsis. Exogenous treatments have been reported to influence root growth response in a concentration-dependent manner. HEA-*p*ABA was found to promote earlier root development, when compared with IAA, inhibiting primary root length and promoting the formation of lateral roots. The contrasting biological activities of HEA-*p*ABA and HEA-*p*NBA were explained analyzing the binding modes and affinities of new compounds in relation to IAA into cucumber and tomato TIR1 auxin receptors. The experimental and theoretical results correlate well and highlight HEA-*p*ABA as a fluorescent compound with potential auxin-like activity in all studied species. These promising results will serve as a starting point to extend the utility of docking new auxin compounds by proposing CsTIR1 and SlTIR1 beside AtTIR1 as working models in understanding the auxin mode of action. Additional to phenotypic observation of root development and auxin-binding activities, a future challenge remains to explore the expression levels of auxin-responsive genes such as ARFs (Auxin-response factors), SAURs (Small auxin-up RNAs) and GH3s (Gretchen Hagen 3). However, the development of new sustainable fluorescent auxin-like compounds and understanding their underlying mechanism of action in plants deserve further investigations.

## Figures and Tables

**Table 1:** The influence of different concentration treatments on seed germination.

Germination (%)									
	Arabidopsis			Cucumber			Tomato		
Treatment	0.1 mM	0.5 mM	1 mM	0.1 mM	0.5 mM	1 mM	0.1 mM	0.5 mM	1 mM
Control	100 ^a^	100 ^a^	100 ^a^	98.66 ± 1.53 ^a^	98.66 ± 1.53 ^a^	98.66 ± 1.53 ^a^	100 ^a^	100 ^a^	100 ^a^
HEA-*p*ABA	98.00 ± 0.86 ^a^	95.11 ± 1.66 ^b^	93.33 ± 1.25 ^b^	94.21 ± 2.29 ^b^	91.96 ± 2.63 ^b^	90.22 ± 2.96 ^b^	88.89 ± 1.36 ^b^	91.11 ± 1.66 ^b^	93.33 ± 1.2 ^b^
HEA-*p*NBA	96.56 ± 2.63 ^b^	89.33 ± 2.96 ^c^	87.66 ± 2.03 ^c^	88.56 ± 2.88 ^c^	87.23 ± 2.49 ^b^	82.00 ± 3.02 ^c^	86.67 ± 2.73 ^b^	71.11 ± 2.49 ^c^	75.56 ± 2.3 ^c^
IAA	77.66 ± 2.49^c^	-	-	86.65 ± 2.27 ^c^	80.45 ± 2.63 ^d^	73.89 ± 3.22 ^c^	75.56 ± 2.03 ^c^	-	-

The significant differences among the variants noted with different letters are based on one-way ANOVA, Fisher LSD (*p* < 0.05).

## Data Availability

Not applicable.

## Supplementary Materials

The following are available online at https://www.mdpi.com/1422-0067/22/6/2797/s1, Figure S1: Root growth phenotype of *C. sativus*, *S. lycopersicum* and *A. thaliana* under different treatments and concentrations, Figure S2: Anion overlay of HEA-*p*ABA and HEA-*p*NBA, Figure S3: Overlay of CsTIR1 and SlTIR1 homology models. Overlay between crystallized and docked conformation of IAA in CsTIR1 and SlTIR1 binding sites, Figure S4: Overlay of IAA, HEA-pABA and HEA-pNBA in the auxin binding site of CsTIR1 and SlTIR1, Table S1: Sequence similarity and identity of CsTIR1 and SlTIR1 with AtTIR1, Table S2: Interactions of IAA in AtTIR1, CsTIR1, and SlTIR1 binding sites.
