# Supplementary material for: Bioprospecting Fluorescent Plant Growth Regulators from Arabidopsis to Vegetable Crops
Source: Int J Mol Sci. 2021 Mar 10;22(6):2797. doi: 10.3390/ijms22062797 (PMC7999160; doi:10.3390/ijms22062797)
Supplement: Supplementary file 1 [file ijms-22-02797-s001.pdf]

Supplementary material

Bioprospecting fluorescent plant growth regulators from Arabidopsis to vegetable crops

Radu L. Sumalan [1], Liliana Halip [2,*], Massimo E. Maffei [3], Lilia Croitor [4], Anatoli Siminel [4], Izidora Radulov[5], Renata M. Sumalan[1] and Manuela E. Crisan[2,*]

2.1. Bioactivity Assays

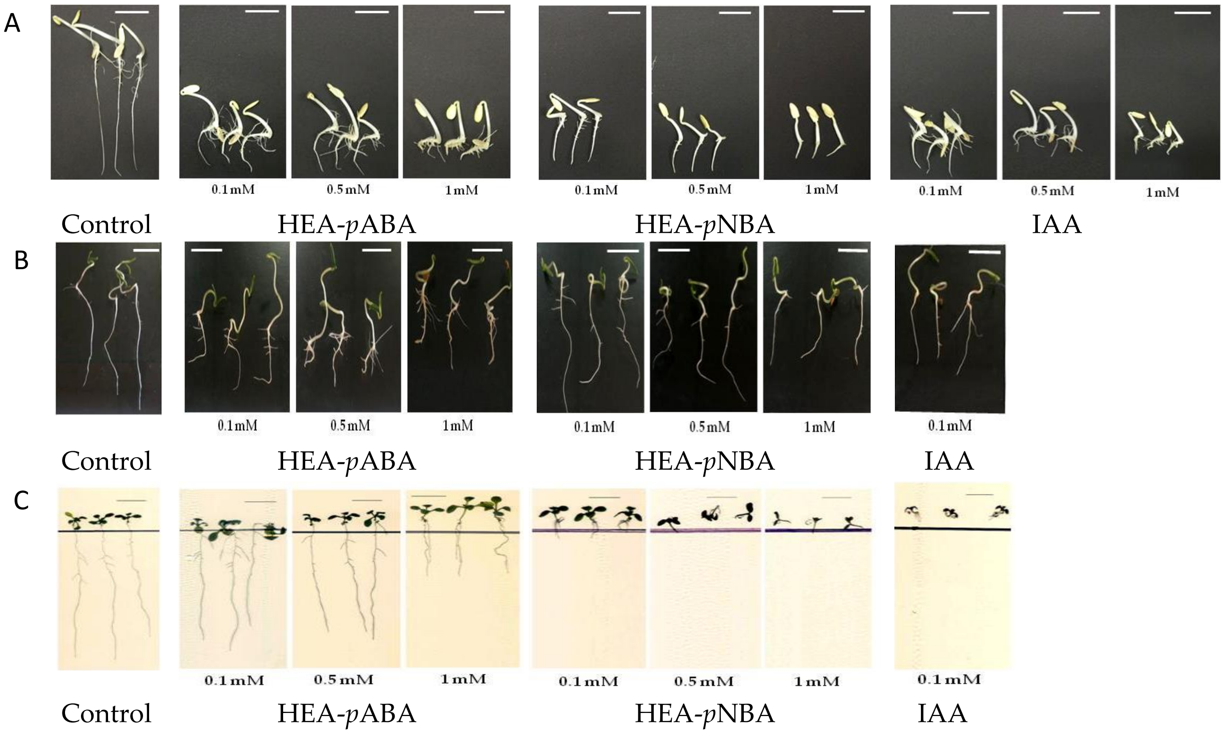

Figure S1. Root growth phenotype of *C. sativus* (A), *S. lycopersicum* (B) and *A. thaliana* (C) under different treatments and concentrations. IAA completely inhibited the germination when used at 0.5 mM and 1 mM in tomato and Arabidopsis. Scale bar, 1 cm.

2.3. Hirshfeld Surfaces

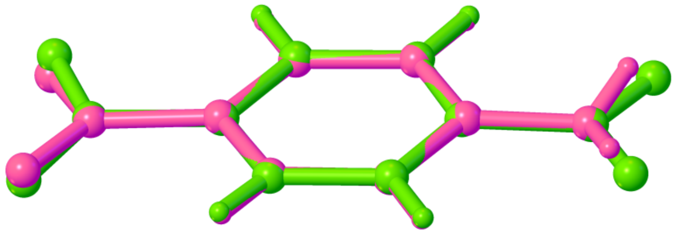

Figure S2. Anion overlay of HEA-*p*ABA (pink) and HEA-*p*NBA (green). The carbon atoms of phenyl rings are coincident in each.

2.4. Homology Modeling and Ligand Docking

Table S1. Sequence similarity and identity of CsTIR1 and SlTIR1 with AtTIR1

Sequence	SSfs[1] (%)	SIfs[1] (%)	SSbs[1] (%)	SIbs[1] (%)
CsTIR1	75.5	58.6	93.3	86.7
SlTIR1	90.8	78.6	100	93.3

[1] SSfs: sequence similarity with AtTIR1 based on full sequence; SIfs: sequence identity with AtTIR1 based on full sequence; SSbs: sequence similarity with AtTIR1 based on binding site sequence; SIbs: sequence identity with AtTIR1 based on binding site sequence;

Table S2. Interactions of IAA in AtTIR1, CsTIR1, and SlTIR1 binding sites

Interaction type (functional group from ligand)	AtTIR1	CsTIR1	SlTIR1
Salt bridge (COO-)	Arg403; Arg 436	Arg431; Arg398	Arg399; Arg432
Hydrogen bond (COO-)	Arg406; Ser438	Arg398; Ser433	Arg399; Ser434
Hydrogen bond (NH)	Leu439	Leu434	Leu435
π- alkyl (pyrrole ring)	Cys405	Cys400	Cys401
π - alkyl (phenyl ring)	Ala464	Ala459	Ala460
π - cation (phenyl ring)	Arg489	-	-

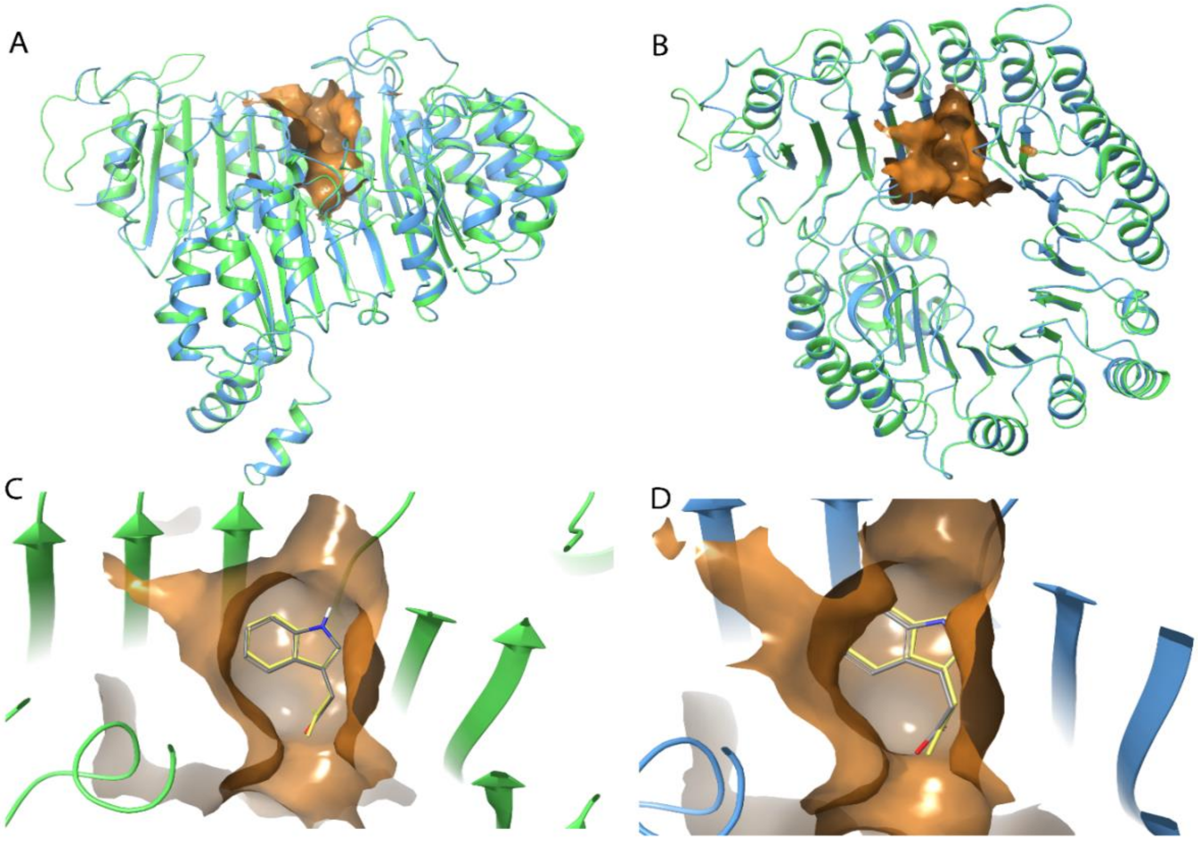

Figure S3. Overlay of CsTIR1 (green) and SlTIR1 (blue) homology models: front (A) and top (B) view. Overlay between crystallized (yellow) and docked conformation of IAA in CsTIR1 (C) and SlTIR1 (D) binding sites (orange surface)

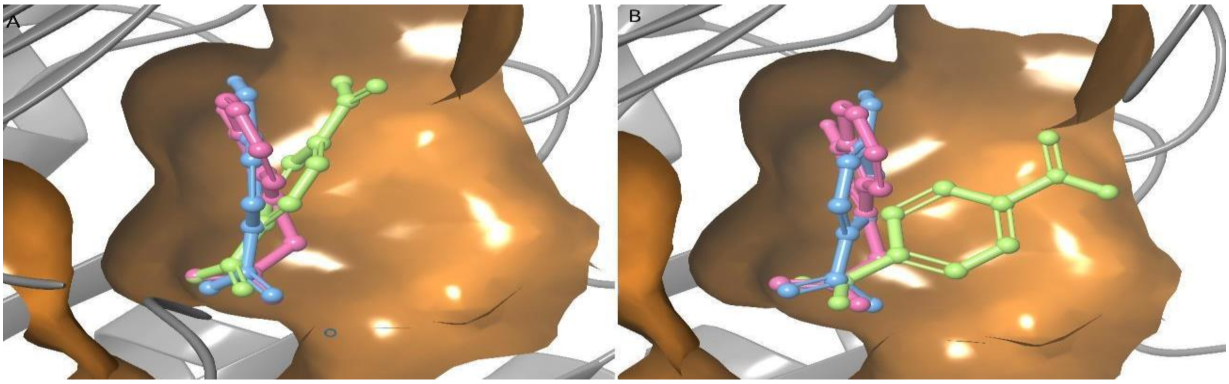

Figure S4. Overlay of IAA (pink), HEA-*p*ABA (blue) and HEA-*p*NBA (green) in the auxin binding site of CsTIR1 (A) and SlTIR1 (B).
